# Computational Simulation of Colorectal Cancer Biomarker Particle Mobility in a 3D Model

**DOI:** 10.3390/molecules28020589

**Published:** 2023-01-06

**Authors:** Esteban Vallejo Morales, Gustavo Suárez Guerrero, Lina M. Hoyos Palacio

**Affiliations:** 1 Grupo de Investigación Sobre Nuevos Materiales, Universidad Pontificia Bolivariana, Medellín 050031, Colombia; 2 Grupo de Investigación e Innovación en Energía, Institución Universitaria Pascual Bravo, Medellín 050036, Colombia; 3 Grupo de Investigación en Biología de Sistemas, Universidad Pontificia Bolivariana, Medellín 050031, Colombia

**Keywords:** colorectal cancer, biomarker particles, early detection, in situ

## Abstract

Even though some methods for the detection of colorectal cancer have been used clinically, most of the techniques used do not consider the in situ detection of colorectal cancer (CRC) biomarkers, which would favor in vivo real-time monitoring of the carcinogenesis process and consequent studies of the disease. In order to give a scientific and computational framework ideal for the evaluation of diagnosis techniques based on the early detection of biomarker molecules modeled as spherical particles from the computational point of view, a computational representation of the rectum, stool and biomarker particles was developed. As consequence of the transport of stool, there was a displacement of CRC biomarker particles that entered the system as a result of the cellular apoptosis processes in polyps with a length lower than 1 cm, reaching a maximum velocity of $3.47\times 10^{-3}$ m/s. The biomarkers studied showed trajectories distant to regions of the polyp of origin in 1 min of simulation. The research results show that the biomarker particles for CRC respond to the variations in the movements of the stool with trajectories and speeds that depend on the location of the injury, which will allow locating the regions with the highest possibilities of catching particles through in situ measurement instruments in the future.

## 1. Introduction

**Figure 1:**
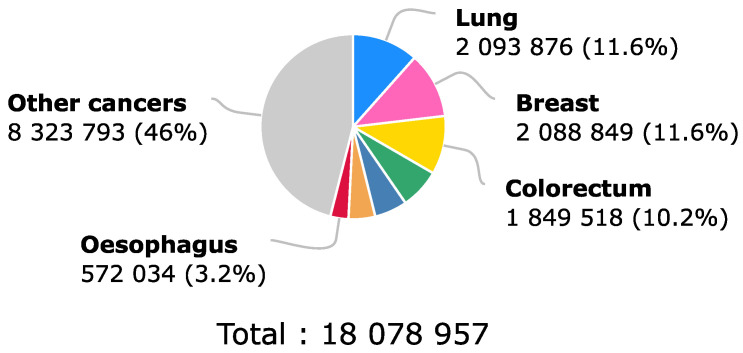
Estimated number of new cancer cases in 2018 worldwide for both sexes and all ages. Here, 33% of the incidence of cancer in the world population during 2018 corresponded to lung, breast and colorectal cancer, which was in third place, which is why the impact of better early diagnosis methods would help a significant portion of the population. Recovered from [1].

**Figure 2:**
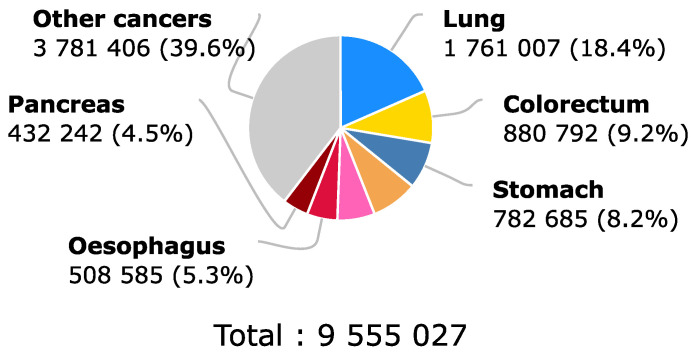
Estimated number of deaths due to cancer in 2018 globally for both sexes and all ages. Colorectal cancer is positioned as the second cancer with the highest mortality during the year 2018. This is due in part to late diagnosis of the disease. Earlier detection could help reduce the 9.2% death rate. Recovered from [1].

According to the World Health Organization [1], and as can be observed in Figure 1, for 2018, colorectal cancer held third place at a worldwide level for the estimated number of new cases in human beings with 10.2 % of the total of new cases, considering both sexes and any age. This number was only surpassed by breast cancer, which was in second place, and by lung cancer, which was in first place on the list. Even though the number of new incidences of colorectal cancer placed it in third place, the same did not happen with the proportion that it represented in the number of deaths in the same year. This can be evidenced by Figure 2, where the number of deaths due to colorectal cancer represented 9.2% of the total deaths caused by cancer in general, positioning colorectal cancer as the second-deadliest cancer in 2018 when considering both sexes and any age [1].

Notwithstanding the reported degree of morbidity and mortality, colorectal cancer generally develops as a result of the neoplastic progression of adenomas to adenocarcinomas, and this can take decades, which enables having an opportune advantage for the early detection of CRC [2] to prevent the complete development of the tumor and improve the prognosis.

With the aim of contributing to the reduction of mortality caused by colorectal cancer, some investigations have focused on improving diagnostic techniques, concentrating their efforts on the identification and sensing of biomarkers associated with the onset of the disease and its corresponding progression.

Even though the study of biomarker molecules in tissues or blood is frequently mentioned by several examples of research [3,4,5,6], there is also a special interest in the biomarker molecules resulting from exfoliation processes in precancerous injuries and CRC where the cell-free DNA (cfDNA) molecules are liberated to the stool, especially in treatments with noninvasive diagnoses [3,7,8,9]. This is because it has been stated that DNA analysis in stool is much more effective than DNA analysis in blood [7].

The first report on DNA hypomethylation about the general contents of 5-metilcitosina in CRC tissues was reported in 1983 by Feinberg and Vogelstein, mentioning that hypo methylation is accompanied by hypermethylation and transcriptional silencing of tumor suppression genes or genes that codify DNA repair proteins [10].

The detection of colorectal neoplasms through a stool DNA test is naturally facilitated by several biological factors, among them being an imbalance in the intestinal microbiota, especially in some bacteria that cause the induction of carcinogenesis through mutations in cells or epigenetic changes that promote cell growth [11], while on the other hand, the unproportioned copious exfoliation and the functional surface area become notably large in neoplasias [12].

Regarding research in the field of computational simulation, most of the studies reviewed focused on the modeling of nanostructured biosensors [13,14,15], showing mathematical models and the results of simulations of the characteristics of the biosensor without specifically mentioning their application in the early detection of diseases. On the other hand, there were studies found on cancer evolution models based on agents and implementing high-performance computing (HPC) [16,17], as well as the progression of melanoma [18], evolution of metastatic processes, tumor angiogenesis [16], treatment response [19,20] and simulation of the adherence of therapeutic particles [21], evidencing efforts that are much more oriented toward the treatment and description of the evolution of CRC, which is oriented toward the early diagnosis of the description of the behavior of biomarkers in a dynamic domain.

Despite this, Tóth Kinga et al. [22] published a research article with the objective of obtaining quantitative data to explore possible correlations between the presence of the biomarker mSEPT9 in the tissue and in the plasma of the same patients, especially in patients with adenoma. There was not any research found that completely coincided with the focus of this work, which describes the behavior of colorectal cancer biomarker molecules that are released by tumors in the processes of cell apoptosis, and it focuses on demonstrating that these particles respond to the movements of feces with trajectories and speeds that depend on the location of the injury, which will help on-site test tools find the best places to capture particles.

## 2. Results

A 3D computation model of the rectum was created using ANSYS^®^ software [23], and it enabled us to simulate the interaction between two peristaltic type II waves, the stool and the CRC biomarker molecules, which were modeled as spherical particles from the point of view of computational simulation. The model was tested for different positions of polyps within the domain of this study, showing different behaviors in the function of the location of the particle exfoliation site.

### 2.1. Behavior of the Stool during the Passing of the Contraction Waves

**Figure 3:**
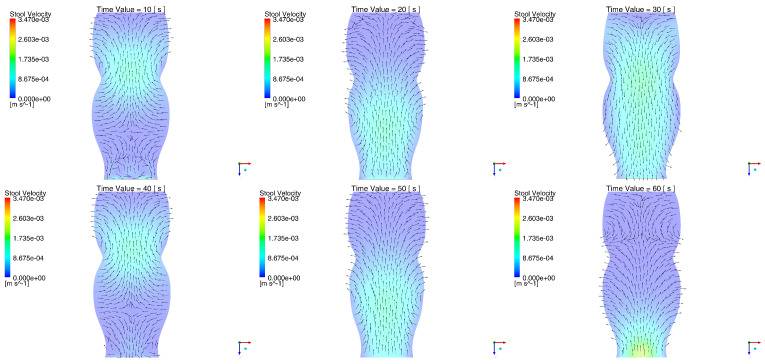
Stool velocity during the type II waves between 10 s and 60 s. The fecal velocity field can be seen in the anterograde and retrograde directions, which implies consistency with the physical behavior described by the fecal mass after the passage of a peristaltic wave.

Figure 3 identifies the field of speeds generated by the contraction of the rectum wall due to the passing of the type II wave for 10≤t≤60 s. The encounter between the retrograde and anterograde speeds can be appreciated in the viscous material between 10 and 60 seconds, while for the tiles of 20 s, 30 s and 50 s, there was a field whose components in –z showed a uniform reverse of the stool. At t = 40 s (and other times), a divergent behavior could be appreciated in the lower part of the domain, where the higher portion showed a retrograde direction and the lower portion was directed toward the exit border of the domain. The maximum velocity of the stool reached by the simulations corresponded to $3.47\times 10^{-3}\;m/s$. The following are the speeds for two of the times that evidenced projection in the trajectory of the stool coming from the region of liberation of the PT 4 biomarker particles.

**Figure 4:**
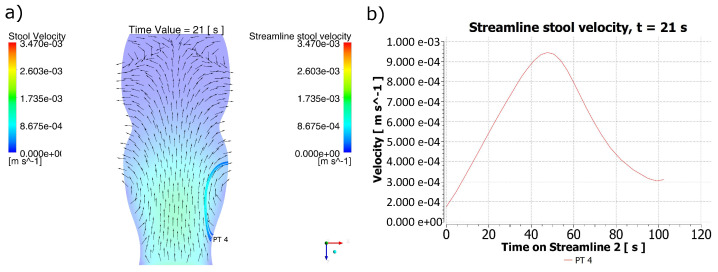
Behavior of the stool for t = 21 s. (**a**) Stool flow lines from the region of the polyp PT 4 for t = 21 s. (**b**) Velocity for the stool flow lines. It can be identified that in the PT4 particle release area, the direction of the fecal mass formed a retrograde trajectory. However, due to changes in the position of the peristaltic contraction, there was a variation in the magnitude of the maximum velocity reached during a new predicted trajectory, indicating a region much further from the point of origin of the biomarker particles.

For t = 21 s in Figure 4a, a retrograde trajectory of the stool was detected coming from the cfDNA PT 4 exfoliation region, and its changes in velocity can be appreciated in Figure 4b, where there is an evident acceleration toward half of the path and a decrease in velocity as it finished its trajectory. According to this figure, the time required for the displacement of the material from the PT 4 point toward the reached region was 102 s.

**Figure 5:**
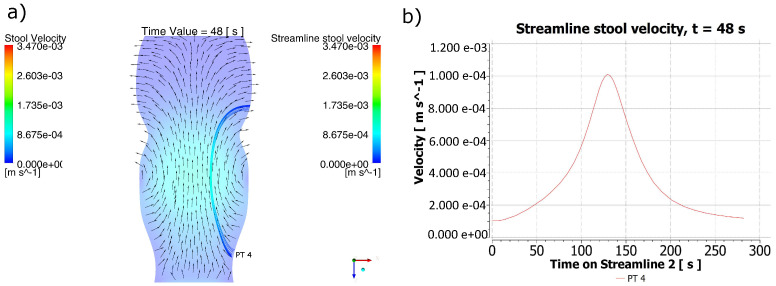
Behavior of the stool for t = 48 s. (**a**) Stool flow lines from the region of the PT 4 polyp for t = 48 s. (**b**) Velocity for the stool flow lines. It can be identified that in the PT4 particle release area, the direction of the fecal mass formed a retrograde trajectory. However, due to changes in the position of the peristaltic contraction, there was a variation in the magnitude of the maximum velocity reached during a new predicted trajectory, indicating a region much further from the point of origin of the biomarker particles, which allows us to state that the direction of the trajectory is retrograde for the type of contraction contemplated, but the site of impact with the predicted colonic wall varies depending on the position of the contraction.

For t = 48 s in Figure 5a, a retrograde trajectory of the stool was detected coming from the cfDNA PT 4 exfoliation region, and its changes in speed can be appreciated in Figure 5b. Since the highest speeds of the stool were at a higher distance from the PT 4 polyp, the maximum velocity reached by the current line corresponds to a time higher than that in Figure 4b. Notwithstanding this, the possibility of reaching regions farther from the polyp is evident for this distribution of speeds. The time required to reach the region of destiny with the speeds obtained for t = 48 s was 281.88 s.

### 2.2. Behavior of Biomarker Particles during the Passing of the Contraction Waves

**Figure 6:**
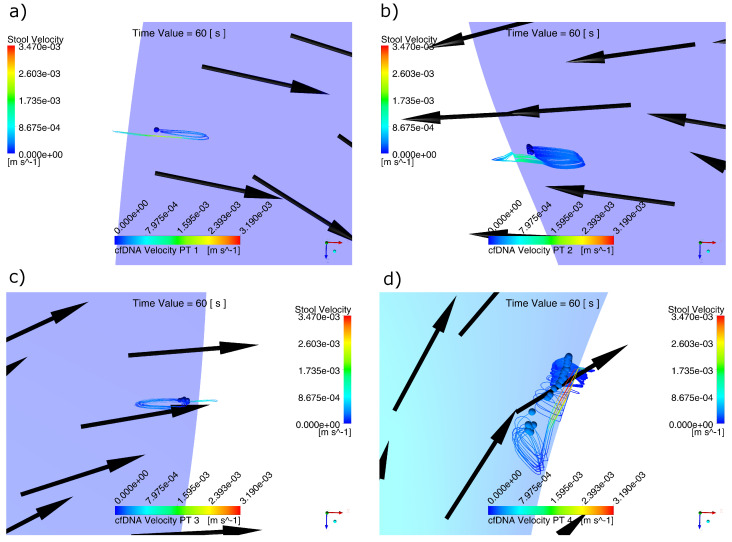
Behavior of the cfDNA particles for t = 60 s: (**a**) region PT1, (**b**) region PT 2, (**c**) region PT 3 and (**d**) region PT 4. The behavior of almost circular trajectories with an advance in the retrograde direction can be explained by the constant change in the direction and magnitude of the velocity field generated by the peristaltic wave.

The computational simulation of coupled domains enabled us to obtain the behavior of the cfDNA modeled as spherical particles in four different liberation zones. In Figure 6, the trajectories of the particles liberated in the regions established for the studied polyps can be identified.

Due to the anterograde and retrograde directions of the field of speeds of the stool, the biomarker particles liberated exhibited a trajectory similar to an ellipsis. Such behavior is especially visible in Figure 6d, since its displacement direction obeyed to the velocity vectors of the stool corresponding to t = 60.

**Figure 7:**
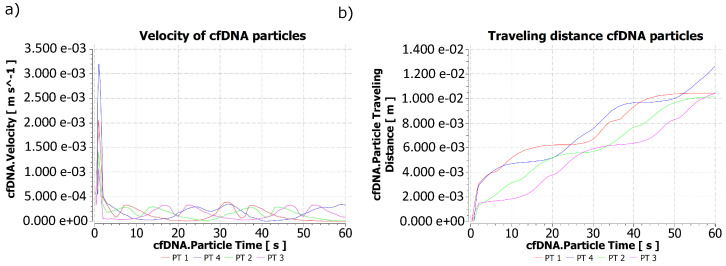
Behavior of the cfDNA particles during 60 s of simulation: (**a**) Velocity of cfDNA particles and (**b**) Traveling distance of cfDNA particles. When observing the line that represents each particle in different places of a colon sample, it is possible to identify the reason why some particles did not seem to have a linear traveled distance, since fluctuations in the velocity magnitude during the simulated time could slow down or accelerate each of the studied particles.

The maximum velocity obtained by the biomarker particles corresponded to $3.19\times 10^{-3}$ m/s, which oscillated in value according to the behavior of the medium that contained them. Figure 7a shows the results of the velocities obtained by the computational simulation.

Figure 7b identifies the traveling of the particles in each region of liberation over 60 s. The maximum travel distance reached by a cfDNA particle in the time of the simulation corresponded to $1.27\times 10^{-2}$ m for the PT 4 injection region, while the region whose particle liberation showed the lowest travel distance corresponded to $1.01\times 10^{-2}$ m for PT 2, with this being a significant distance in relation to the size of the particle and the time that passed.

After comparing the changes in velocity of the particles in the PT 4 region obtained in Figure 7a and the position of the maximum velocities reached by the stool for t = 10, 20, 30, 40, 50 and 60 s, an increase and decrease could be identified in the velocities of the biomarker particles in proportion to the increase and decrease in the stool velocity, which evidences a successful coupling between the study domains.

## 3. Discussion

This research was able to identify the behavior of the biomarker particles of colorectal cancer at an early stage through a dynamic and similar model to the real biological conditions. By using the interaction of coupled domains, it was possible to identify the behavior of the cfDNA liberated into the stool under functionality conditions, because the dynamics of the model was generated by contraction waves whose parameterization came from clinical studies on the magnitude of pressures generated on the colon’s motility.

The incorporation of the peristaltic waves as the transportation mechanism for the rectum contents enabled us to obtain the velocities of the stool and biomarkers since, with this mechanism, it was guaranteed that the movement obtained was as a consequence of the colon’s motility.

In the dissemination process of the biomarker particles through the stool, there was verification that such the analyte moved to distant regions of the injury in only one minute of simulation. The strategic location of an on-site diagnosis device would enable higher effectiveness in the catchment of the analyte, since the predictive nature of the simulation showed those zones of the intestinal tract where stool would eventually arrive with cfDNA particles, and since the results were in terms of the trajectory, velocity and travel distance for the biomarker particles, future studies can use these findings, as the parameters calculated for the construction of on-site biomarker measurement instruments maximized the capitation of cfDNA particles.

The inclusion of elements that were not considered in other investigations [13,14,15,16,21] allowed us to obtain a more adjusted model in terms of the functionality conditions. Some of the results concurred with behaviors that have already been described in other studies, while others enabled us to identify values that did not correspond with the results reported by other authors due to their simplifications.

Determining the trajectories of the biomarkers enables the development of new noninvasive and moderately invasive diagnosis and treatment techniques, increasing the control of the development of cancer in morbid persons and achieving higher effectiveness in treatments for people suffering from colon cancer, which corroborates the usefulness of computational simulations in medical diagnoses as a tool for the solution of complex problems, whose analytic solution is difficult, unknown or impossible to obtain.

Due to the lack of in vivo clinical techniques for the simultaneous observation of biomarker nanoparticles and the stool in a colon in motion, it was necessary to build mathematical models to obtain the parameters necessary for the simulation, which became a contribution for future research. In the same manner, due to the lack of in vivo experimental results, the results obtained in this research were analyzed in light of similar computational simulations, supporting most of the considerations taken since they supported their simplifications.

## 4. Materials and Methods

Computational implementation was performed using Ansys CFX software [23]. Its meshing tools were used, as well as its ability to perform mechanical and fluid analysis, incorporating the discrete phase to represent the release of colorectal cancer biomarkers.

### 4.1. Material Properties: Rectum Wall

The hyperelastic approach postulates the existence of a function of W energy, which relates the traveling of the colon’s tissue with the corresponding tension values. The deformation energy function represents the energy stored by a system under deformation. When the load is removed, the deformation energy is gradually liberated into the system in such a manner that it returns to its original form [24].

A homogeneous and isotropic material was assumed to represent the rectum walls, with the argument from several works that models of the colon’s behavior toward contractions caused by peristaltic movement or toward interaction with a colonoscopy should use these simplifications [24,25,26].

For a homogeneous material, the deformation energy is the only function of the deformation gradient *F*, defined as

(1) $$F=\frac{\partial x}{\partial X}.$$

where *x* denotes a point in the current configuration and *X* denotes a point in the reference configuration. For isotropic materials, the deformation energy W=W(F) is also a function of the invariants $I_{1}$, $I_{2}$ and $I_{3}$, which are defined as follows:

(2) $$I_{1}=trace\left(C\right)=\lambda_{1}^{2}+\lambda_{2}^{2}+\lambda_{3}^{2}.$$

(3) $$I_{2}=\frac{1}{2}trace\left(C\right)=\lambda_{1}^{2}\lambda_{2}^{2}+\lambda_{1}^{2}\lambda_{3}^{2}+\lambda_{2}^{2}\lambda_{3}^{2}.$$

(4) $$I_{3}=\lambda_{1}^{2}\lambda_{2}^{2}\lambda_{3}^{2}.$$

where $\lambda_{1}$, $\lambda_{2}$ and $\lambda_{3}$ are the stretching coefficients and *C* is defined as the Cauchy–Green deformations tensor, which represents the deformation measure of a point in a direction *i* and has the products of the deformation gradients. This tensor is determined through the following equation:

(5) $$C=F^{T}F$$

The colon tissue is incompressible; that is, the volume of the material remains constant during the deformation. For incompressible materials, the following is true:

(6) $$det\left(C\right)=\lambda_{1}^{2}\lambda_{2}^{2}\lambda_{3}^{2},$$

making the deformation energy a function of only two invariants:

(7) $$W=W(I_{1},I_{2}).$$

There are various mathematical models for representing hyperelastic isotropic materials, including the neo-Hookean, Arruda–Boyce, Gent, Mooney–Rivlin, Ogden, polynomial, Yeoh and Ogden approaches. Polynomial forms are most popular for the constitutive modeling of biological tissues due to their simplicity and, therefore, efficiency in the calculation processes [27,28].

**Figure 8:**
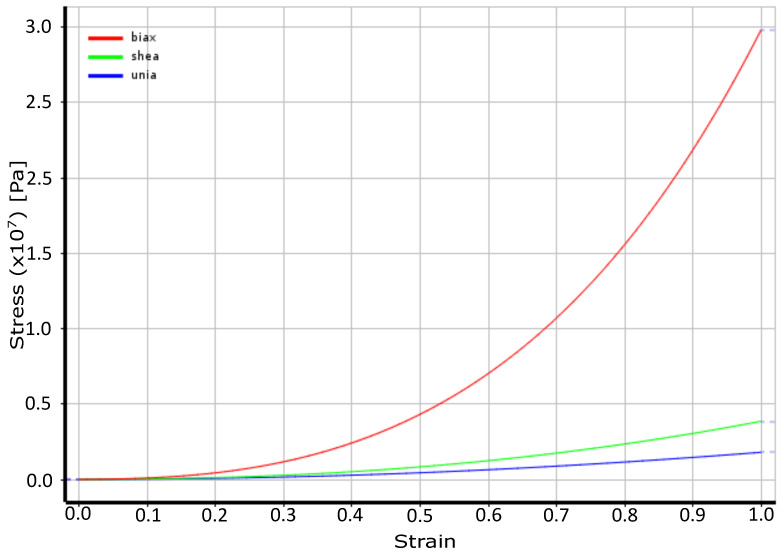
Behavior of the material according to the three-parameter Mooney–Rivlin model.

In this work, the Mooney–Rivlin three-parameter model was used as in [29], whose behavior can be appreciated in Figure 8. The mathematical model corresponding to the Mooney–Rivlin three-parameter formulation consists of the following equation:

(8) $$W=C_{10}\left(I_{1}-3\right)+C_{01}\left(I_{2}-3\right)+C_{11}\left(I_{1}-3\right)\left(I_{2}-3\right)+\frac{1}{d}{\left(J-1\right)}^{2}$$

where $C_{10}$,$C_{01}$ and $C_{11}$ are the material rigidity constants, *d* is the incompressibility parameter and *k* is the volume module.

Since for an incompressible material $J=\lambda_{1}\lambda_{2}\lambda_{3}=1$, *W* can be simplified as follows:

(9) $$W=C_{10}\left(I_{1}-3\right)+C_{01}\left(I_{2}-3\right)+C_{11}\left(I_{1}-3\right)\left(I_{2}-3\right)$$

The work of Xuehuan [24] enabled the correct parameterization of the model through the values given in Table 1.

According to Mcintosh and Anderson [30] in their work A Comprehensive Tissue Properties Database Provided for the Thermal Assessment of a Human At Rest, the density of the human colon corresponds to the value $\rho =1132\;\mathrm{kg}/m^{3}$.

The incompressibility parameter *d* could be found based on the following equation:

(10) $$d=\frac{1-2\nu }{c_{10}+c_{01}}$$

This value was obtained from the Poisson coefficient reported in [31] and the optimized constants reported in Table 1. Its value corresponded to $d=4.60\times 10^{-7}$.

Figure 8 illustrates the tension deformation obtained with the three-parameter Mooney–Rivlin formulation.

### 4.2. Peristaltic Movement

In adults, the rectum has a resting pressure of 6mmHg (799.934Pa) [32,33] and shows three motion patterns:

1. Type I contractions: simple monophasic waves of low width and short duration. These contractions form holes on the surface creation pressures of 5 to 10 cm of $H_{2}O$ (490.333–980.665 Pa), their duration varies from 5 to10 s, and their frequency is 8 to 12/min [34].
2. Type II contractions: These have a greater width 8, 15 to 30 $\mathrm{cm}\;H_{2}O$ (784.532, 1471, 2942 Pa), and last longer (25 to 30 s), their frequency is 2/min; both contractions act to mix the stool [34].
3. Type III contractions: These represent a change in the base pressure, generally lower than 10 $\mathrm{cm}\;H_{2}O$ (980.665 Pa), with superposition of type I and II waves [34].

**Figure 9:**
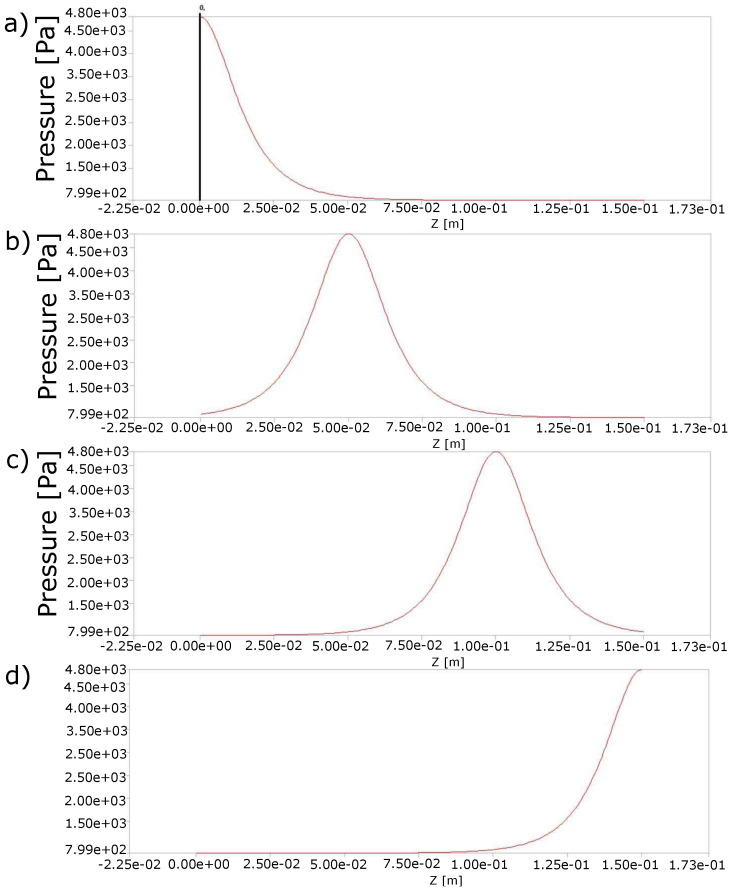
Travel of the peristaltic wave on the z-axis over time: (**a**) 0 s, (**b**) 10 s, (**c**) 20 s and (**d**) 30 s, which were repeated for another 30 s after passing the second wave. Changes in pressure as a consequence of muscle contractions contributed to the transport of the fecal mass and with it the biomarker particles that were released in the process of cell apoptosis.

The domain $\Omega_{s}$ was defined to note the structure formed by the colon walls. Such a structure is deformed by a contraction wave perpendicular to the external surface of the geometry that travels through the longitude of the rectum on the z-axis. The contraction wave that mimics the muscular contraction of the peristaltic movement is represented in Figure 9 and is given by

(11) $$p\left(z,t\right)=p_{0}+p_{1}sech\left[\frac{b_{1}\left(z-ct\right)-b_{2}}{L}\right]$$

where $b_{1}$ and $b_{2}$ are constant as well as $p_{0}$ and $p_{1}$, which are the resting pressure and active pressure, respectively [35], and *L* is the length of the chosen colorectal section. The constant c represents the velocity of the wave, whose value corresponding to 1 cm/s [36] is adapted to 0.5 cm/s in order to reach the frequency of the type II waves (2 waves/min) mentioned in [34].

Equation (11) can also be expressed as

(12) $$p\left(z,t\right)=p_{0}+p_{1}\left\{\frac{2}{e^{\frac{b_{1}\left(z-ct\right)-b_{2}}{L}}+e^{-\frac{b_{1}\left(z-ct\right)-b_{2}}{L}}}\right\}$$

The active pressure was determined to be $p_{1}=3999.67\;\mathrm{Pa}$, which was in the range of pressures reported in the colon and the rectal sigmoid section [37,38], and the rest pressure was $p_{0}=\left(799.934\;\mathrm{Pa}\right)$ [32,33]. The length of the rectum was $L=1.50\times 10^{-1}\;m$. The values for the remaining parameters were $b_{1}=14$ and $b_{2}=0$ for the first wave and $b_{2}=2.1$ for the second wave.

### 4.3. Stool

The importance of this domain in the research is because of it being the medium in which the cfDNA biomarker particles are liberated. Such particles respond to the changes in velocity generated by the interaction of the peristaltic movement of the colon walls and the stool under study.

“The stool is made up of proteins, fats, fiber, bacterial biomass, inorganic materials, and carbohydrates. Its chemical and physical characteristics vary widely depending on the person’s health and diet” [39]. Nevertheless, for practical effects, parameters such as the density and viscosity of the stool were taken from the research conducted by R. Penn et al. [39] and corresponded to $1060\;\mathrm{kg}/m^{3}$ and 5.50Pas, respectively.

For this research, the stool was modeled as a highly viscous incompressible fluid with Newtonian behavior through the conservation laws of Navier–Stokes through Equation (15), supported by the argument that there was analysis in one section of the colon destined for storage, and therefore, most of the changes in the viscosity were in sections prior to the rectal section. In addition, “The length of the intestine modeled is considerably reduced (15 cm), restricting the possible variation of the viscosity” [40].

The flow was described in terms of the Cauchy tensor [41,42,43]:

(13) $$\overset{=}{T}=2\mu D\left(\vec{u}\right)-pI$$

where: μ is the viscosity, *p* is the pressure and $\bar{\bar{D(\vec{u})}}$ is the deformation tensor [41,42,43], a term that can be expressed as

(14) $$\bar{\bar{D(\vec{u})}}=\frac{1}{2}\left(\nabla \vec{u}+\nabla^{T}\vec{u}\right)$$

The fluid dynamics can be described by a transitory domain through the expression

(15) $$\rho \left(\frac{\partial \vec{u}}{\partial t}+\left(\vec{u}\cdot \nabla \right)\vec{u}\right)-\mu \Delta \vec{u}+\nabla p=\vec{F}$$

We considered the condition of the incompressible fluid as follows:

(16) $$\nabla \cdot \vec{u}=0$$

where: ρ and μ are the density and viscosity of the flow, respectively, $\vec{F}$ represents the exterior forces and $\vec{u}$ is the Euler velocity of the fluid. This model was valid only if the fluid was viscous (μ>0).

The first two terms in Equation (15) refer to the inertial mass forces of the fluid. The third term describes the viscous forces of the fluid, while the pressure gradient expressed in the fourth term represents the pressure forces, and the fifth term refers to the external forces. In such equation, the expression $\left(\left(\vec{u}\cdot \nabla \right)\vec{u}\right)$ is known as the convective non-linear term, what contributes to the difficulty to reach the convergence of the solution.

For the problem studied, the temperature considered was 37 °C (310.15 K) and invariable in time and space.

The terms $\Omega_{f}$ and $\Gamma_{f}$ were defined to represent the domains of the fluid and its borders, respectively. The border conditions imposed were as follows:

- A Dirichlet-type condition for the fluid velocity at the entry border given by
  (17) $$\vec{u}=0\;\;\;\mathrm{over}\;\;\;\Gamma_{f}$$
- A Neumann condition given by
  (18) $$\overset{=}{T}\cdot n=\vec{\sigma }\;\;\;\mathrm{over}\;\;\;\Gamma_{f}$$

This carried the velocity gradients $\vec{u}$ through the fluid tension tensor and showed a pushing effect over the edge of the fluid.

### 4.4. Mesh

**Figure 10:**
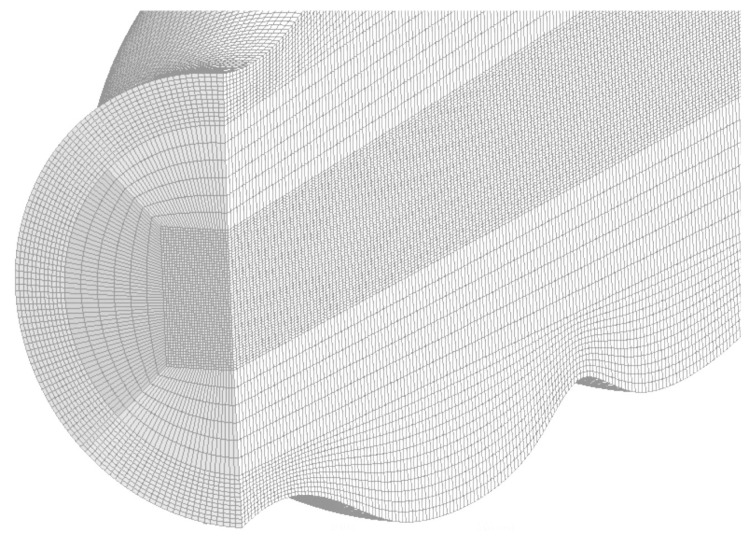
Structured mesh of the rectal lumen model. This type of structured meshing contributes significantly to the stability and convergence of the solution.

**Figure 11:**
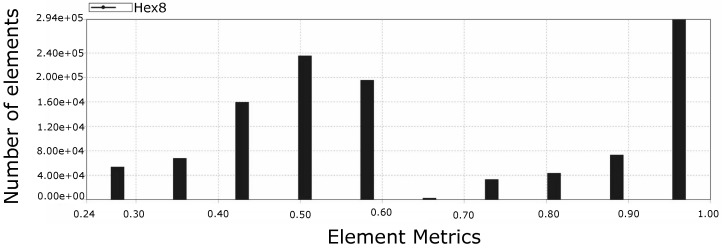
Mesh quality report of rectal lumen model, where 0 is poor element quality and 1 is perfect element quality. The predominance of elements with a metric of 1 contributed to the success of the simulation and generated confidence in the results obtained. Note: Hex8 is the type of element used by the mesh and represents a hexahedron with 8 nodes.

**Figure 12:**
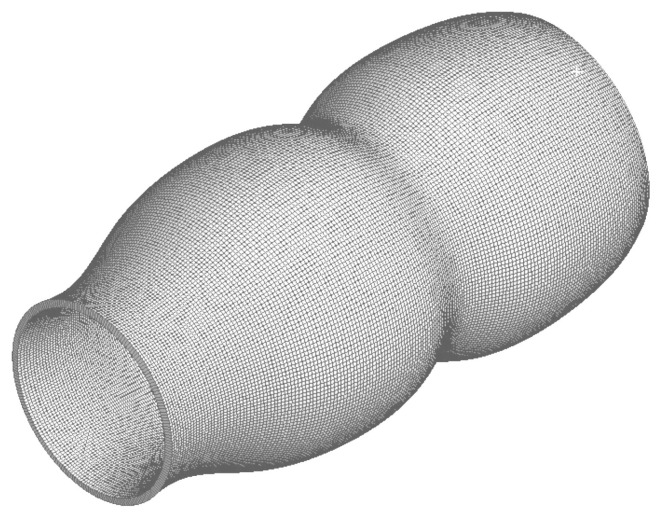
Structured mesh of the rectal wall portion of the colon.

**Figure 13:**
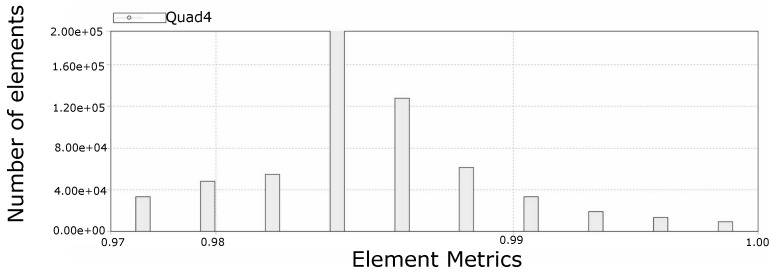
Mesh quality of the rectal wall portion, where 0 is poor element quality and 1 is perfect element quality. The absence of elements with a quality lower than 0.97 accounted for an adequate reticulation of the colon wall, which allowed adequately representing the deformation of the material. Note: Quad4 is the type of element used by the mesh and represents a quadrilateral with 4 nodes.

Figure 10 presents a section that allows visualizing the structured mesh generated for the lumen of the colon portion studied, while Figure 11 reports the quality of the mesh in Figure 10. On the other hand, Figure 12 and Figure 13 refer to to the mesh associated with the colon wall and its corresponding quality report respectively.

### 4.5. Description of the Biomarker Particles

Circulating extracellular DNA (cfDNA) is present in the blood or liberated in the lumen of the large intestine. The amount of cfDNA circulating in serum and plasma seems to be significantly higher in patients with tumors than in healthy controls, especially for those with tumors in advanced states compared with tumors in early stages. Samanta Salvi et al. [44] carried out several studies destined to correlate the reordering in tissue and plasma samples, which coincided to confirm that the analysis of circulating DNA can be used as a diagnosis tool. It has been suggested that cfDNA in healthy people is mainly of a hematopoietic origin. Nevertheless, cfDNA in cancer patients also results from apoptotic and necrotic processes characteristic of tumor cells with high cellular refill. Apoptosis produces DNA fragments of approximately 180 base pairs (bp) or corresponding multiples, while necrosis produces much larger fragments [9,44,45,46].

According to the studies performed in the field of DNA biomarkers in colorectal cancer, an average molecular weight is commonly accepted for each base pair of DNA of $1.02\times 10^{-24}$ kg. According to several studies [9,44,46], the cfDNA fragments have an amount of base pairs that ranges from 115 to 247 base pairs (bp) [9,45,47]. By taking 200 bp as a recurrent value in such texts, it was determined that the mass of dfDNA particles was $2.04\times 10^{-22}$ kg.

According to [48], the length between base pairs is used to find the total length of a DNA fragment, since it is mentioned that for a length of 2000 bp, the theoretical length is 680 nm. Supported by the data of such research, it was determined that for 200 bp, the length was 68 nm. Such a calculated value was used to model the size and form of the particle, assuming that cfDNA is a sphere whose diameter corresponds to 68 nm. A spherical shape was assumed for the 3D particle, so the volume of a sphere representing a cfDNA particle was $9.26\times 10^{-23}\;m^{3}$.

Once the dimensions and mass of a cfDNA particle were clear, it was determined that, for this study, the density of a fragment of a DNA biomarker for colorectal cancer corresponded to approximately $\rho \approx 2.20\;\mathrm{kg}/m^{3}$.

Table 2 summarizes the mass, density and dimensions of the particles.

#### 4.5.1. Colon Epithelial Cells

The epithelial cells of the intestines have been very difficult to cultivate in vitro as primary cells. Due to this, over the last four decades, the preferred model of the intestine epithelium has been transformed, having CaCo-2 cells as the main reference, whose composition and behavior are particularly adequate [49].

According to Jung et al., it has been estimated that in patients with colon cancer and a tumor of 100 g, close to 3.3 % of the tumor’s DNA is liberated daily in the blood flow [45].

The volume of a CaCo-2 cell is $V=1.40\times 10^{-15}$ m^3^. Assuming that all enterocytes are diploid, their DNA content is $5.70\times 10^{9}$ base pairs per cell [50,51]. According to the results found in [52], the mass of a cell is $3.00\times 10^{-12}$ kg. Such magnitudes have been estimated based on the similarities between enterocytes and CaCo-2 cells validated through [49], and they were used in this research for the calculation of the diffuse flow of cfDNA particles.

#### 4.5.2. Exfoliation Processes

Neoplasias abundantly exfoliate the dysplastic cells and their components in the colon, granting a regular supply of analyte for stool analysis. Since direct histological observation has stated that such exfoliation seems to be continuous [12], such results were used as the starting point to assume a constant injection flow of cfDNA toward the the colon for this model.

The reason why the analysis of the cfDNA liberated toward the stool showed much more significant results for the early detection of CRC than those obtained in the tests of blood hidden in feces is the fact that hidden blood is the result of circulation through a hemorrhage, which is intermittent and often absent in precancerous injuries [12].

#### 4.5.3. Calculation of Parameters for the Injection of Particles through the Rectum Wall

In order to adequately define the injection of cfDNA particles, certain parameters need to be calculated. In this section, a mathematical model is built for the calculation of mass flow, the amount of particles injected per second and the magnitude of the injection velocity of the particles.

**Figure 14:**
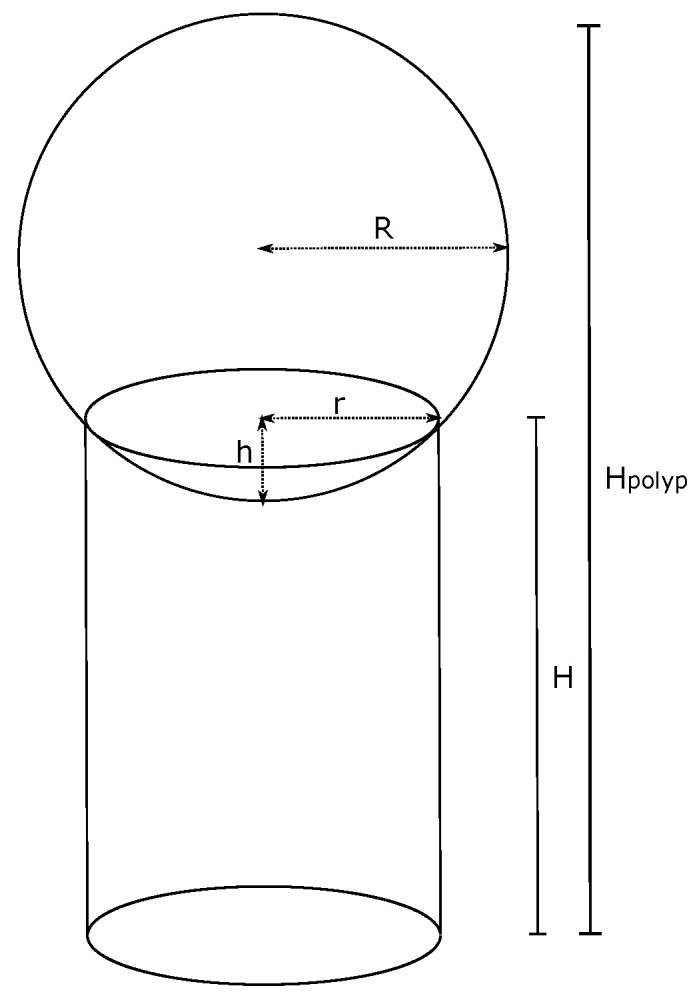
Simplified representation of a neoplastic polyp of the tubular adenoma type.

For the calculation of the mentioned parameters, it was required to establish a geometric model that represented the structure of the polyp in a simplified manner. Figure 14 represents a neoplastic polyp of a tubular adenoma type, which is made up of a height cylinder *H* representing the stem and a sphere with a radius *R* representing the higher part of the polyp. The height of the structure $H_{polyp}$ was used to calculate the total area through which a detachment of DNA fragments from the cells that made up such a structure was produced.

The area of the Figure 14 was calculated based on the following thought:

A sphere is defined and located over the higher face of the height of a cylinder $H=3.00\times 10^{-3}$ m and with a base radius of $r=1.50\times 10^{-3}$ m. The intersection between both figures generated a spherical cap with a radius $r=1.50\times 10^{-3}$ cm and with a height of *h*. A total height $H_{polyp}=7.00\times 10^{-3}$ m was defined for the complete structure, so the diameter of the sphere was $D_{s}=7.00\times 10^{-3}-3.00\times 10^{-3}+h$ m; that is, $D_{s}=4.00\times 10^{-3}+h$ m, and therefore, its radius was $R=\frac{D_{s}}{2}$.

The total area of the polyp corresponded to the addition of the area of the spherical portion and the stem, obtaining $A_{polyp}=8.67\times 10^{-5}$ m^2^ as a result.

The volume of the proposed polyp can be calculated as follows:

(19) $$V_{polyp}=\left(V_{sphere}-V_{cap}\right)+V_{stem}$$

where

(20) $$V_{sphere}=\pi r^{2}H$$

(21) $$V_{cas}=\frac{\pi h(3r^{2}+h^{2})}{6}$$

Therefore, after performing such calculations, it was found that $V_{polyp}=1.42\times 10^{-5}$ m^3^.

If the volume of the polyp was divided into the previously reported volume of a CaCo-2 cell, the result was the amount of cells that made up such a polyp. With such a calculation, the total mass of the proposed polyp could be estimated under the supposition that such neoplasia was totally made up of only CaCo-2 cells:

(22) $$PolypCellAmount=\frac{V_{polyp}}{V_{CaCo-2}}=1.02\times 10^{10}$$

Therefore, the mass of the polyp was 0.03 kg.

Once the area of the polyp was calculated, it was necessary to determine the velocity for the injection of particles into the system. Due to the specific nature of the data, and after an extensive review of the pertinent literature, it was found that such a value was not explicitly reported in the research dedicated to analysis of the exfoliation of biomarker particles in a neoplastic polyp. As an answer to such a question, a previous simulation without the injection of particles was proposed in order to determine the minimum velocity reached (different from 0 m/s) by part of the stool after a peristaltic movement with a duration of 15 s in the rectum. The results in terms of the velocity were exactly the same in the presence or absence of particles since, for the interest of this study, and also considering the size of the particles, the effect that these had on the simulated viscous mass was not taken into account.

After an evaluation of the average velocity reached for each time step in the simulation, the research proceeded with the representation of such data in time, the exportation of the results and selection of the minimum value. Once this was performed, it was determined that the velocity assigned at the entrance of the particles to the system was $5.57\times 10^{-4}$ m/s.

With all the parameters identified, the mass flow was defined as

(23) $$\dot{M}=\frac{\Delta M}{\Delta t}=\rho \frac{\Delta V}{\Delta t}=\rho A_{polyp}\left|\vec{v}\right|$$

where ρ is the density expressed in Table 2 and *v* is the entrance velocity stipulated for the injection of particles.

For a magnitude with a particle entry velocity $v=5.57\times 10^{-4}$ m/s, the mass flow would then be $\dot{M}=0.06\times 10^{-6}$ kg/s.

The mass flow and particle size were considered to calculate the real (physical) number of particles. It was assumed that each numerical particle was a group of real particles that behaved in the same manner. In order to know how many particles hid behind the numerical particles, the mass flow and sizes must be supplied.

The particle flow rate was defined as

(24) $$T_{fp}=\frac{\dot{M}}{M_{cfDNA}}=2.99\times 10^{14}\;particles/s$$

where $M_{cfDNA}$ is the mass of a cfDNA particle, expressed in Table 2.

Nevertheless, such a value is very elevated and requires an excessive amount of resources from the system. Therefore, such a value was divided by the amount of particles intended to be represented within only one particle that represented them graphically.

To obtain the parameters associated with the diffusion of biomarkers in the stool, the information supplied by Jung et al. was used, who estimated that in patients with colon cancer with a tumor size of 100 g, approximately 3.3% of the tumor’s DNA was liberated daily into the blood flow [45]. Given that, in the literature reviewed, there was no explicit explanation of an amount for a polyp of 0.03 kg, the percentage reported by Jung et al. was used to establish a linear relation and estimate the percentage of cfDNA liberated daily for the studied neoplasia as follows:

(25) $$cfDNA_{percentage}=\frac{0.03\;\mathrm{kg}\times 0.03}{0.10\;\mathrm{kg}}=0.99\%$$

This percentage of cfDNA was analyzed as follows.

An average molecular weight of $1.02\times 10^{-24}$ kg is commonly accepted for each base pair (bp) of DNA [53]. Given that a cell contains $5.70\times 10^{9}$ bp, the mass of the cfDNA present in a cell within a polyp of 0.03 kg could be calculated as follows:

(26) $$M_{DNA}=5.70\times 10^{9}\;\frac{\mathrm{bp}}{\mathrm{cell}}\left(1.02*10^{-24}\;\frac{\mathrm{kg}}{\mathrm{bp}}\right)=5.81\times 10^{-15}\;\frac{\mathrm{kg}}{\mathrm{cell}}$$

If the liberation of cfDNA was assumed to be continuous during one day (86,400 s), and 0.99% of the tumor’s DNA was liberated daily in the blood flow, then the flow of liberated cfDNA could be calculated as follows:

(27) $$\varphi =5.81\times 10^{-15}\;\frac{\mathrm{kg}}{\mathrm{cell}}\left(1.01\times 10^{10}\;\mathrm{cell}\right)\left(\frac{0.99\;\%}{\;\mathrm{day}}\right)\left(\frac{1\;\mathrm{day}}{86400\;s}\right)=6.72\times 10^{-12}\;\frac{\mathrm{kg}}{s}$$

Finally, according to Fick’s first law, the density of diffusive flow is expressed as

(28) $$J=\frac{\varphi }{A_{polyp}}$$

Therefore, we have

(29) $$J=\frac{6.72\times 10^{-12}\;\frac{\mathrm{kg}}{s}}{8.67\times 10^{-5}\;m^{2}}=7.79\times 10^{-8}\frac{\mathrm{kg}}{m^{2}s}$$

## 5. Conclusions

This research contributes new knowledge to advance the search for solutions to the early detection of colorectal cancer with treatments that can decrease morbidity. The development of mathematical and computational models implemented to solve biological situations, especially in cases where there is interaction between macroscopic and nanoscopic mediums, such as in this research, offers opportunities for the generation of new developments that can be used to analyze other cases of the same disease or other diseases, which contributes to the implementation of solutions for the monitoring of cfDNA concentrations in real time, since this can be one of the most effective techniques for the early diagnosis of CRC, given that diverse studies highlight the diagnostic and predictive importance of the analysis of biomarkers in the stool.

The lack of scientific information on some parameters that were characterized and used computationally in this research became an opportunity to provide mathematical models to calculate their values. This contributed to appropriate mathematical modeling and computational implementation, and there were findings that showed similarities with a real biological situation, what increased the reliability of the results.

The future possibility of implanting a biosensor coupled to the rectum for patients with risk factors would represent a great advantage in terms of a reduction in the time required for the collection and analysis of the samples, as well as the preparations necessary for the following colonoscopies.

It is concluded that this research will be useful for future studies associated with the early diagnosis of colon cancer, due to the multidisciplinary approach to the phenomenon, the computational simulation tools and the mathematical modeling based on scientific evidence that supported its development.

Finally, this research is a computational study in which there is no experimental evidence. The validity of the findings obtained must be verified based on references from other future research oriented toward the analysis of biomarkers in diseases.

## Figures and Tables

**Table 1:** Rigidity constants of the isotropic hyperelastic material.

Constant	Value (Pa)
$C_{10}$	652.01
$C_{01}$	42,835.25
$C_{11}$	219,120.30

**Table 2:** Characterization of the biomarker particles.

Length (bp)	Length (nm)	Mass (kg)	Volume ($m^{3}$)	Density ($\mathrm{kg}/m^{3}$)
200	68	$2.04\times 10^{-22}$	$9.26\times 10^{-23}$	2.20

## Data Availability

Not applicable.
